# Materials Design and Structural Health Monitoring of Horizontal Axis Offshore Wind Turbines: A State-of-the-Art Review

**DOI:** 10.3390/ma18020329

**Published:** 2025-01-13

**Authors:** Yihui Tong, Weitao Liu, Xuanyi Liu, Peng Wang, Zhe Sheng, Shengquan Li, Hao Zhang, Yuwei Meng, Ye Zhu, Xubing Lei, Ying-Tien Lin, Pengcheng Jiao

**Affiliations:** 1 Zhejiang Energy Digital Technology Co., Ltd., Hangzhou 310000, China; 22334098@zju.edu.cn (Y.T.); liuweitao@zjenergy.com.cn (W.L.); liuxuanyi@zjenergy.com.cn (X.L.); mengyuwei@zjenergy.com.cn (Y.M.); 2 Hainan Institute, Zhejiang University, Sanya 572024, China; 22234137@zju.edu.cn (S.L.); hao.zhang@zju.edu.cn (H.Z.); kevinlin@zju.edu.cn (Y.-T.L.); pjiao@zju.edu.cn (P.J.); 3 CGN Zhejiang Daishan Offshore Wind Power Co., Ltd., Zhoushan 316200, China; wangpeng880384@163.com (P.W.); szzs0017@163.com (Z.S.); 4 Marine Monitoring and Forecasting Center of Zhejiang Province, Hangzhou 310000, China

**Keywords:** Offshore Wind Turbines (OWTs), material design, structural health monitoring (SHM)

## Abstract

In recent decades, Offshore Wind Turbines (OWTs) have become crucial to the clean energy transition, yet they face significant safety challenges due to harsh marine conditions. Key issues include blade damage, material corrosion, and structural degradation, necessitating advanced materials and real-time monitoring systems for enhanced reliability. Carbon fiber has emerged as a preferred material for turbine blades due to its strength-to-weight ratio, although its high cost remains a barrier. Structural Health Monitoring Systems (SHMS) play a vital role in detecting potential faults through real-time data on structural responses and environmental conditions. Effective monitoring approaches include vibration analysis and acoustic emission detection, which facilitate early identification of anomalies. Additionally, robust data transmission technologies are essential for SHMS effectiveness. This paper reviews material design strategies, data acquisition methods, and safety assessment techniques for OWTs, addressing current challenges and future directions in the field.

## 1. Introduction

Over recent decades, as science and technology have rapidly advanced, Offshore Wind Turbines (OWTs) have become integral to the clean energy transition, significantly benefiting both social and economic spheres [1]. The two common types of wind turbines are Vertical Axis Offshore Wind Turbines (VAOWTs) and Horizontal Axis Offshore Wind Turbines (HAOWTs), where VAOWTs are mainly used for small scale applications to generate electricity in urban areas or remote areas that are not connected to the grid. Horizontal Axis Offshore Wind Turbines (HAOWTs), which are the most commonly used type of wind turbine, are more technologically advanced, capable of generating more power output and have a wider application at sea [2]. Therefore, this paper explores the damage and health monitoring of offshore horizontal axis wind turbines. Operating in dynamic and often harsh marine conditions, OWTs face critical safety challenges, including blade damage, material corrosion, structural degradation, electrical system failures, and foundation instability. Such issues not only disrupt turbine operations but also pose significant safety risks [3]. Thus, utilizing advanced materials, implementing innovative structural designs, and employing real-time monitoring systems are essential strategies to enhance the long-term stability and performance of OWTs [4]. Among these components, turbine blades—central to energy generation—require materials with a high strength-to-weight ratio to withstand the significant bending forces generated by strong winds [5,6]. In recent years, carbon fiber has gained favor over traditional glass fiber due to its superior strength and lightweight characteristics, emerging as the material of choice for blade construction [7]. However, the high cost of carbon fiber remains a major challenge to its broader adoption [8]. Additionally, the structural foundations of OWTs must endure complex environmental loads, including forces from wind, waves, sea ice, and tides. While gravity-based and monopile structures are commonly used in shallow waters, the move to deeper offshore locations necessitates the development of floating structures, which are rapidly becoming an industry standard. To extend turbine lifespan, there is also a growing focus on developing advanced anti-corrosion technologies for both blades and supporting structures [9].

The rapid advancement of sensing technology in recent years has positioned Structural Health Monitoring Systems (SHMS) as a pivotal technology for ensuring the long-term reliability of Offshore Wind Turbines (OWTs) [10]. Using advanced sensor technologies, SHMS continuously gathers real-time data on both structural response and environmental conditions, facilitating the early detection of potential faults and enabling comprehensive condition assessments. Common monitoring approaches include vibration analysis, acoustic emission detection, and strain measurement [11,12]. Vibration monitoring employs accelerometers and gyroscopes to capture data on vibrations induced by wind and wave forces, allowing for the identification of structural anomalies through modal analysis [13]. Acoustic emission detection focuses on high-frequency stress waves, which can reveal the early formation of internal cracks, providing critical early warnings of potential failure. Strain measurement is used to assess the performance of key components—such as the tower, blades, and foundation—under complex loads, helping engineers to optimize design and reduce maintenance costs. Additionally, robust data transmission systems are integral to SHMS effectiveness [14]. Wireless technologies like LoRa, Wi-Fi, NB-IoT, and Zigbee, which outperform traditional wired methods in harsh marine environments, offer enhanced reliability and long-distance transmission capabilities, contributing to improved safety and cost-efficiency for OWT operations [15].

To ensure the operational and structural safety of Offshore Wind Turbines (OWTs), researching load identification and safety assessment methods during their normal operation phase is essential [16,17]. Over their operational lifetime, OWTs are subject to a range of complex loads, including aerodynamic wind forces, wave loads, and harmonics such as 1 P and 3 P loads, which directly impact the stability of the structure [18]. Precise identification of these loads is essential, typically achieved through both time-domain and frequency-domain methods. Time-domain approaches estimate loads based on acceleration and strain data, though they can be prone to noise and cumulative errors [19]. Frequency-domain methods, on the other hand, utilize inverse transfer function models to derive dynamic loads, although they encounter limitations near the structure’s natural frequency [20]. Conducting comprehensive safety assessments that integrate finite element models with vibration response data has proven effective for long-term damage detection and monitoring, thereby enhancing the operational safety of OWTs.

Existing research on offshore wind turbine Structural Health Monitoring (SHM) systems in the literature mainly focuses on specific aspects such as material and structural design, data acquisition and transmission, and safety assessment methods. However, a comprehensive study of SHM systems for Offshore Wind Turbines is lacking. Web of Science database and Google Scholar were the primary sources for evaluated publications and research outcomes. This review search focuses on the existing literature on advanced materials and innovative structural designs for offshore wind turbines, data acquisition and transmission for structural health monitoring systems, load identification and safety assessment. The search covered the period from 2000 to 2024 and consisted mainly of journal articles published in English, which also included some review papers. However, it is inevitable that some of the more relevant articles from before 2000 were found during the search, and this is disclaimed. In addition, the search was not limited to specific regions or countries but was conducted globally. The main screening process was carried out in two steps. In the first step, we conducted an initial screening on Web of Science with the main key aspects of offshore wind turbine health monitoring: “offshore wind turbine”, “structural health monitoring”, “vibration analysis”, ”fatigue damage detection”, and “materials design”, which includes some journal articles and review papers. In the second step, the introductory and methods sections of the screened literature were further reviewed to ensure that the studies met the inclusion criteria and were strongly related to our topic. By screening the literature, we finally selected 182 papers for inclusion.

**Figure 1:**
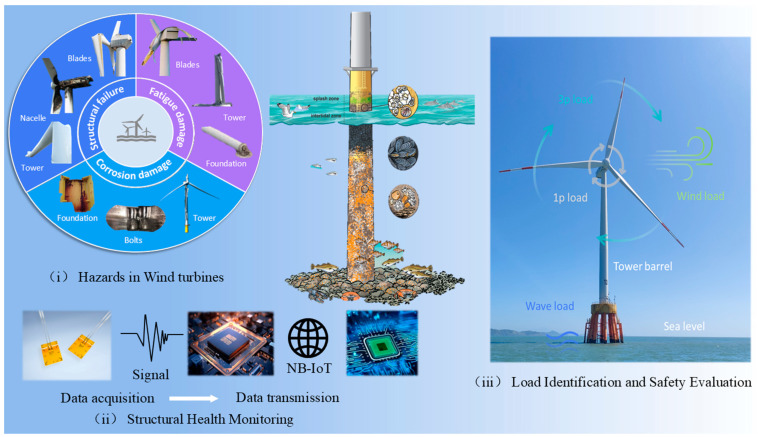
Graphical abstract—From wind turbine hazards to structural health monitoring to load identification and safety evaluation.

The presented review articles talk about the structural health monitoring aspects in wind turbines. A brief description, contribution, and limitations can be seen in Table 1. Several review articles provide comprehensive insights into the structural health monitoring (SHM) of offshore wind turbines (OWTs). These reviews cover various aspects, including current monitoring methods, technologies, challenges, and future directions. In contrast to the above review, this paper covers the material property requirements of key OWT components, highlighting the trade-offs between cost, weight, and strength. It also examines the role of SHM systems in detecting failures and ensuring long-term reliability, focusing on vibration analysis, acoustic emission detection and strain measurement. In addition, the paper discusses load identification and safety assessment methods for optimizing OWT design and operation. Overall, the review emphasizes the importance of integrating advanced materials, innovative structural designs and real-time monitoring systems to improve the operational safety and performance of offshore wind turbines. Figure 1 shows a graphical representation of this work and a complete interactive extension, thus ensuring the novelty of this comprehensive review.

**Figure 2:**
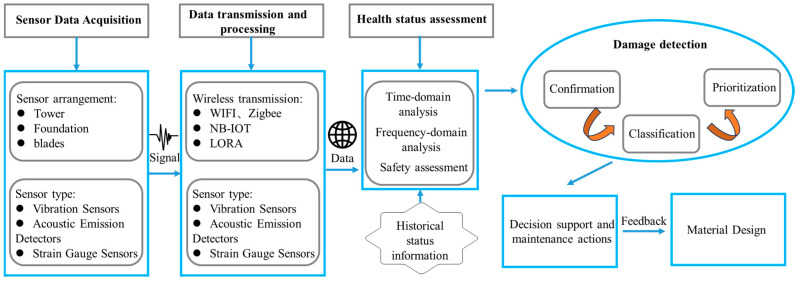
Overall flow chart for offshore wind structure health monitoring.

The paper is structured as follows: Section 2 reviews material design strategies for key SHM components of OWTs; Section 3 details data acquisition and transmission technologies, offering a comparison of four widely adopted wireless transmission methods; Section 4 examines load identification and safety assessment approaches typically used during OWTs’ operational phase; and Section 5 outlines the current challenges and future directions in SHM for Offshore Wind Turbines. Figure 2 shows the overall process of offshore wind structure health monitoring, from data acquisition to fault diagnosis and maintenance. The illustration can include steps such as sensor data acquisition, data transmission and processing, health status assessment, damage detection, decision support and maintenance actions.

## 2. Material Design for OWTs

### 2.1. Material Performance Requirements

#### 2.1.1. Rotor Blades for OWTs

**Figure 3:**
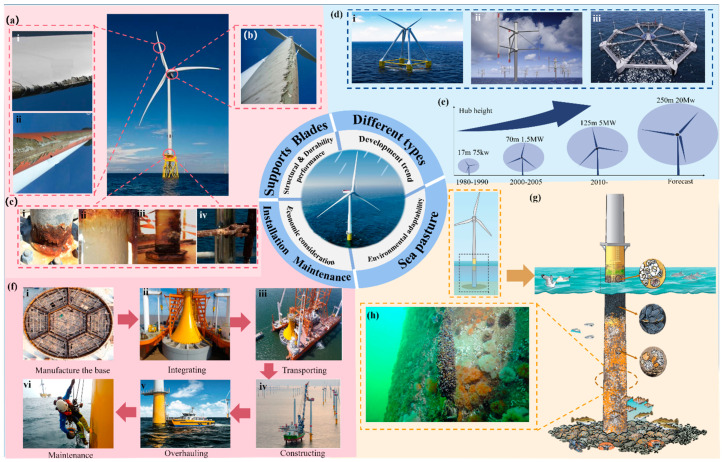
A summary of losses, costs, and development trends related to OWTs. (**a**) Leading-edge erosion of wind turbine blades [35]. (**i**) Development of leading-edge cracks. (**ii**) Shedding of the surface layer. (**b**) Rain erosion of wind turbine blades [36]. (**c**) Different types of erosion on support structures [37]. (**i**) Uniform or general corrosion. (**ii**) Pitting corrosion. (**iii**) Stress-corrosion cracking. (**iv**) Corrosion fatigue. (**d**) Different types of OWTs: (**i**) A pyramid-shaped floating wind turbine with the nacelle supported by four masts [38]. (**ii**) A new vertical-axis wind turbine designed for offshore applications [39]. (**iii**) An integrated floating platform for Offshore Wind Turbines [40]. (**e**) Wind power blade size development [41]. (**f**) The process of OWTs from construction to maintenance [41,42,43,44]. (**i**) Manufacture the base. (**ii**) Integrate the parts. (**iii**) Transporting the parts. (**iv**) Constructing the OWT. (**v**) Overhaul the OWT. (**vi**) Maintenance the OWT. (**g**) Offshore wind farm structures support invertebrates and fouling organisms, attracting predator fish, seabirds, and marine mammals [45]. (**h**) Biofouling community on an offshore gravity-based wind turbine [45].

Rotor blades are large, monolithic hybrid structures incorporating not only bolt-connected blade-hub joints but also integrated lightning protection features, and utilizing various composite materials and components [27]. Over the past two decades, OWT blades have grown larger in response to increasing turbine power [28]. To control costs while enhancing performance, Franco et al. [29] propose a technique for optimizing OWT blade designs in smart rotors to maximize power output irrespective of wind conditions. The most significant loads experienced by both onshore and offshore wind turbine blades are flap-wise and edgewise bending loads. These flap-wise loads are primarily carried by the blade’s spar, while edgewise loads are supported by the shell structure [30]. Jackson et al. [31] conducted experimental studies and simulations on a small-scale horizontal-axis wind turbine, improving blade shapes by measuring vortices influence generated at the blade edges. Meanwhile, manufacturers are refining blade materials, with composite materials offering substantial value due to their excellent “mass-to-stiffness” ratio [32]. To meet the demands of new types of turbine blades, innovative technologies are necessary. Traditionally, blades are almost entirely made of glass fiber and polymer composites [33]. However, compared to glass fiber, carbon fiber has advantages in reducing the weight of wind turbine blades. Research indicates that new carbon fiber composites from the textile industry might be more suitable for the wind energy sector [34]. Figure 3 presents a summary of losses, costs, and development trends associated with Offshore Wind Turbines (OWTs).

#### 2.1.2. Support Structures for OWTs

Support structure technology for OWTs is an indispensable part of offshore wind technology development [46]. The fabrication and erection of support structures constitute approximately 30% of the overall capital expenditure for wind farms [47]. Thus, selecting the appropriate support structure and their fabrication and installation techniques is critical for OWTs projects. With the increasing size of wind turbines, the support structures must ensure stability and applicability under Significant and intricate loads produced by waves, wind, sea ice, and tides [48]. Various support structures have been designed for different environmental conditions, including gravity-based, monopile, tripod, braced frame, tension leg with suction buckets, floating systems, and so on [49,50,51].

Gravity-based foundation structures were commonly used in early offshore wind farms in shallow waters. Their stability is influenced by their own weight and are often constructed using reinforced or prestressed concrete, offering lower production costs, better durability, and fatigue resistance [52]. Monopiles are also a prevalent type of substructure for OWTs, typically made of steel. They are relatively simple to design, with no significant technical limitations, and are convenient to install and transport. However, as turbine size increases, the diameter of monopiles also grows, leading to exponentially higher material and installation costs [53]. Multi-leg substructures, such as tripods, involve driving piles into the seabed with the aid of special guiding frameworks. The substructures are pre-assembled onshore, transported to the final position by vessels, and then lowered and installed onto the piles [54]. They are connected to the piles via grouting and are suitable for areas with uneven soil distribution, significant sediment thickness variations, and shallow foundations. For instance, Taiwan uses friction and rock-socketed high-rise pile cap foundation technology on the Asian bedrock seabed [55].

Fixed structures are not suitable for deep-sea regions, which has led to the development of floating support structures. Floating support structures must provide sufficient buoyancy to support both the wind turbine’s weight and their own weight. Additionally, they have to offer adequate rotational stability and favorable wave response to prevent system capsizing and to handle dynamic loads without damaging the wind turbine [56]. The importance of floating turbine foundations in deeper waters is increasing day by day, making the choice of materials and structures for them even more important. Conventional steel foundations are expensive and prone to corrosion, resulting in high maintenance costs [57]. Reinforced concrete has been proposed as a cost-effective alternative due to its lower cost and better corrosion resistance [58], and prestressed reinforced concrete foundations have shown good stability and safety under a wide range of loads. Floating wind turbines must withstand combined loads from wind, waves, and currents, which can be extreme in magnitude, and the dynamic behavior under these loads requires complex analysis and design to ensure stability and longevity [59]. Ref. [60] proposed different foundation designs, such as semi-submersible platforms and tension-legged platforms (TLPs), to address site-specific conditions and environmental challenges where the stability of these structures is critical. In 2017, the Hywind Pilot Park off the coast of Scotland began operating some 6 MW turbines, marking the first offshore floating wind farm [61]. In summary, we have briefly summarized the losses, costs, and future development trends related to offshore wind turbine construction as illustrated in the following figure.

### 2.2. Material Selection and Design Approaches

#### 2.2.1. Common Materials Used in OWTs

The development of rotor blade materials is closely related to advancements in wind turbine design. For instance, the increasing size of three-blade upwind turbines necessitates the use of lighter materials to reduce gravitational fatigue loads and harder materials to prevent blade impacts with the tower in strong winds [27]. Generally, common materials include glass fiber, carbon fiber, and biological fibers [62]. J.J.E. Teuwen et al. [63] compared fully glass-reinforced bearing blade components, i.e., the spar cap, with fully carbon-reinforced spar caps and glass-carbon hybrid spar caps. They found that the strength of the fully carbon-reinforced spar cap is approximately twice that of the fully glass-reinforced bearing blade component. However, the relative advantage of glass fiber spar caps is their lower cost and ease of material acquisition compared to carbon fiber, which is about three times more expensive. Due to smaller fiber diameters, carbon fibers have less resin content (RMC), leading to denser fiber packing and poorer reliability [27,64]. Biological fibers, such as sisal, flax, hemp, and jute, have the potential to reduce construction costs and environmental impacts, though their potential effects and practical applications require further research [64]. Shah et al. [65] have also demonstrated the potential for flax-based biological fiber-reinforced blades to enhance strength. Currently, research on the recycling of fiber-reinforced composite waste is ongoing, but practical use remains limited, with most research focused on downcycling to other applications [66]. Future research should focus on improving the recyclability of turbine rotors to enhance the recovery rate of composite materials.

Bio-composites have good environmental benefits, including reduced carbon footprint, sustainability and end-of-life degradability. The use of renewable resources such as natural fibers and biopolymers in bio-composites reduces environmental impacts and has good carbon storage potential, making them a promising alternative material for offshore wind power material design [67]. Although bio-composites have fewer negative environmental impacts in most categories, their impacts in terms of eutrophication and ozone layer depletion may be greater compared to conventional materials [68]. The application of bio-composites for offshore wind may also have limitations in terms of mechanical strength and water absorption, which need to be carefully considered when applying them to ensure safety and durability, as well as challenges such as biofouling and corrosion in offshore structures, which may be exacerbated by the use of certain bio-composites. Therefore, more consideration needs to be given to the selection of bio-composites to be used, for example, through certain effective coatings to avoid these problems [69].

Offshore structures are subject to cyclic waves and wind loads, making fatigue failure prevention crucial in the design of offshore wind turbine support structures [70]. Concrete towers, with their high material damping properties, offer greater durability and reduced risk of dynamic failure. Concrete towers ensure reliability and are cost-effective in manufacturing and maintenance [71]. However, in the corrosive marine environment, traditional concrete materials face various durability threats, such as sulfate attacks, during their service life [72]. In offshore areas, towers are often made of reinforced concrete. Research by Mecal indicates that concrete–steel hybrid towers could be economically viable. Although the initial investment in hybrid towers is higher, their greater height provides increased power generation by the turbines, providing a better return on investment [73]. Concrete–steel hybrid towers show good fatigue performance. Ultra-high-performance concrete provides high strength and durability, reducing the fatigue problems commonly associated with pure steel towers [74], and the addition of steel fibers to ultra-high-performance concrete significantly improves its ductility and fatigue resistance, which is critical in preventing the structure from sudden failure under high cyclic loads [75]. Offshore wind turbine support structures are highly susceptible to corrosion, particularly pitting corrosion, which can significantly reduce fatigue life, and although corrosion protection mechanisms are employed, they have a limited service life and therefore require regular maintenance and monitoring, but their maintenance is a more difficult area [76].

The construction cost of foundations accounts for approximately 20% to 35% of the total cost of current offshore wind farm projects. The type and design of offshore wind turbine foundations are heavily influenced by seabed soil characteristics, water depth, wave height, and water flow. Common materials for foundations, similar to those used in tower structures, include various types of steel that depend on the situation. Traditionally, the European standards EN10025 S355 (or EN 10225 S355) are the main structural steel standards used in wind turbine structural design [77].

#### 2.2.2. OWTs Protection and Materials Practices

Throughout the lifecycle of offshore wind farms, blades are subjected to environmental loads, extreme conditions, and fatigue [27]. Therefore, various tests on blade materials are required during design and manufacturing to ensure performance requirements are met. Full-scale structural testing remains the primary method for validating the comprehensive performance of wind turbine blades [78]. Blade testing methods are categorized into static and fatigue (or dynamic) tests. In static tests, loads are applied statically to the blades, typically in the flap-wise and edgewise directions. Overgaard et al. [79] performed full-scale static flap-wise bending tests on 25-m wind turbine blades, combining numerical modeling to analyze interlaminar fracture and material-induced instability under mixed-mode loads. In this regard, fatigue testing is divided into uniaxial and biaxial tests. Flap-wise load and edgewise load uniaxial tests are usually conducted sequentially, while biaxial tests apply both flap-wise and edgewise loads simultaneously [27]. During fatigue testing of a 3 MW full-scale wind turbine blade, Lee and Park [80] identified delamination failure at the blade root based on the principles of the international standard IEC 61400-23. Comparison of measurement data with numerical analysis revealed that blade root end load distribution was significantly altered by the complex geometry between the maximum chord and root. In addition, experiments are conducted to evaluate the erosion of the leading edge of the blade due to airborne particles, such as rain, in order to investigate the material’s susceptibility to corrosion [35].

Support structures must account for environmental loads, particularly in the tower section, which includes torsional loads [81], axial loads, wave loads, and wind loads [82]. Fatigue cracks induced by these loads are a primary mode of structural failure. Additionally, the corrosive processes in the marine environment exacerbate fatigue phenomena. Therefore, fatigue analysis of wind turbine structures necessitates the use of fatigue testing and fracture mechanics [83].

Many currently operational Offshore Wind Turbines are equipped with anti-corrosion kits. Erosion protection for the blades typically involves a thermoplastic tape applied to the leading edge of the outer blade section [84]. These anti-corrosion kits are primarily designed to prevent both internal and external corrosion of the turbine, especially within its nacelle and support structure [85]. The support structures of Offshore Wind Turbines, which require protection, can be categorized based on the degree of corrosion in different areas: atmospheric area, underwater area, splashing area, and seabed area [86]. Among these, steel structure corrosion is most severe within the splash area as a result of the combined influences of seawater and saline air from the marine environment [87]. Currently, primary methods to address this involve enhancing corrosion allowance, implementing cathodic protection, applying coatings, and utilizing spraying methods [88]. In summary, we have briefly summarized the design, materials, and protections related to OWTs as explained in the following table.

### 2.3. Conclusion of Material Design for OWTs

Based on the contents of Table 2, the design considerations, material selection and protective measures for key components of offshore wind turbines are summarized. Mainly in the following four aspects: blade design needs to consider blade thickness, size, and bending load and wind load design and other factors; commonly used materials include glass fiber, carbon fiber and biofiber composite materials, and the use of coatings and metal coatings and other protective measures; nacelle design needs to consider the adiabatic, heat dissipation and other factors; commonly used materials include iron, low alloy steel and high alloy steel, etc. [27,88,89,90]; the tower design needs to consider the load carrying capacity, structural stability and wind load resistance; commonly used materials include steel and concrete, and the use of coatings, metal coatings, corrosion margins and cathodic protection and other protective measures [73,82,89]; foundation design needs to consider the load carrying capacity, vibration damping and corrosion prevention and other factors; commonly used materials include steel and concrete, and the use of coatings and cathodic protection and other protective measures [82,89].

For cathodic protection, its advantages are that it is simple, reliable, applicable to a variety of environments, and it is a more effective protection strategy because of its moderate initial cost and low maintenance cost, but it is mainly affected by environmental conditions [91]. Coating protection method is also a more effective means of protection, and its advantage is that it is more effective in initial protection, but deteriorates over time and affects the environment, and its initial cost is high and maintenance cost is moderate [92]. Considering both cathodic and coating protection together, the choice between them depends on specific project requirements, environmental conditions, and cost considerations, and combining these strategies can provide enhanced protection and greater cost-effectiveness.

The above material design requirements underscore the importance of ensuring the safe and reliable operation of offshore wind turbines in harsh marine environments. In the future, it is necessary to further optimize the material properties, improve fatigue and corrosion resistance, and combine with advanced monitoring technology to achieve full life cycle monitoring and management of key components.

Overall, this chapter provides a comprehensive overview of the critical components of Offshore Wind Turbines (OWTs), focusing on material selection, design methodologies, and emerging trends. It addresses the advancements and challenges associated with rotor blades and support structures. The transition from glass fiber to carbon fiber in blade materials is aimed at reducing weight and enhancing strength, although it introduces challenges related to fatigue and corrosion. Support structures are designed based on environmental conditions, with options including gravity-based foundations, monopiles, multi-leg structures, and floating platforms. The chapter emphasizes the crucial importance of material fatigue resistance and corrosion resistance. It also explores corrosion protection technologies and testing methodologies for both blades and support structures to ensure the durability and reliability of wind turbines under extreme conditions.

## 3. Data Acquisition and Transmission Technology for Structural Health Monitoring (SHM) of Offshore Wind Turbines (OWTs)

### 3.1. Sensor Technologies for SHM of Offshore Wind Turbines

**Figure 4:**
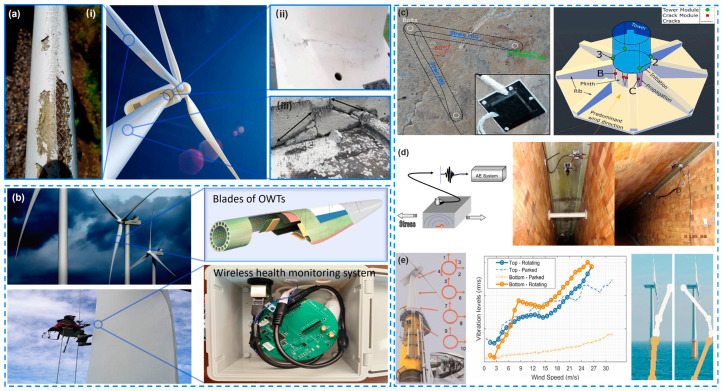
The summary of different sensing techniques used for SHM in OWTs. (**a**) Hazards to which wind turbines may be exposed and where structural health monitoring is required. (**i**) Corrosion on wind turbine blades [116], (**ii**) Cracks in the hub joint area of a wind turbine blade due to fatigue [117], (**iii**) Grout core damage in offshore wind power grout connections [105]. (**b**) From blade damage to manual detection: wind turbine → laminate damage → embedding or attaching SHM sensor-monitoring system → repair. (**c**) Strain measurement techniques for wind turbines [100]. (**d**) Acoustic emission detection techniques for wind turbines [106]. (**e**) Vibration monitoring of Offshore Wind Turbines with 10 sensors in 4 different positions [115,118].

The Structural Health Monitoring System (SHMS) significantly enhances the profitability, reliability, and sustainability of Offshore Wind Turbines (OWTs) through a more systematic operational management approach. It plays a critical role in ensuring the integrity and safety of Offshore Wind Turbines [93]. This technology allows for the acquisition of extensive data from in-situ structures, including structural responses and environmental conditions, facilitating effective fault detection and condition assessment [94]. Various sensor technologies are currently available to continuously monitor the status of these structures for early detection of potential issues. The primary sensing technologies include vibration monitoring, acoustic emission detection, and strain measurement [95]. Table 3 summarizes the comparison of the different monitoring technology for OWTs. Each method has its unique advantages and limitations, making them suitable for different aspects of SHM in offshore wind power structures. Vibration monitoring is best for comprehensive monitoring of blades and foundations, acoustic emission detection excels in impact detection and submerged structures, while strain monitoring is ideal for detailed analysis of tower strain and fatigue. Figure 4 presents a summary of various sensing techniques used for structural health monitoring (SHM) in Offshore Wind Turbines (OWTs).

**Table 3:** The comparison of the different monitoring technology for OWTs.

Technology	Advantages	Limitation	Best-Use Scenarios	References
Vibration monitoring	Early Damage Detection Comprehensive Monitoring Remote Accessibility	Environmental Sensitivity Installation Costs	Blade Monitoring Foundation Monitoring	[96,97,98,99,100]
Acoustic emission monitoring	High Sensitivity Micro-scale monitoring	Complex Signal Interpretation High Sampling Rates Environmental Challenges	monitoring crack propagation, fatigue cracks and material damage	[101,102,103,104,105,106]
Strain Monitoring	Direct Measurement High Accuracy Durability	Installation Complexity High environmental interference Requires multiple locations	Tower Monitoring Fatigue Analysis	[107,108,109,110,111,112,113,114,115]

#### 3.1.1. Vibration Monitoring for OWT SHM

Vibration monitoring is one of the most used methods for OWT SHM. Research by Kim, H.C. [96] and others conducted operational modal analysis of Offshore Wind Turbines, providing robust data and technical support for evaluating turbine safety and ensuring structural design and maintenance. Zhou [97] proposed the deployment of accelerometers at different heights on the turbine to capture vibration data. This technology utilizes accelerometers and gyroscopes to measure vibrations caused by environmental factors, such as wind and waves, as well as the operational status of the turbine. By analyzing vibration data, engineers can identify abnormal patterns that may indicate structural weaknesses, mechanical failures, or fatigue. For instance, a sudden increase in vibration levels could signal impending failures in the turbine’s gearbox or rotor blades [98].

The data collected from vibration sensors can be processed using advanced algorithms to perform modal analysis and identify changes in structural dynamic behavior over time. Studies indicate that this analysis aids in distinguishing normal operational vibrations from those caused by structural anomalies [99].

#### 3.1.2. Acoustic Emission Detection for OWT SHM

Acoustic emission (AE) detection is another valuable technique for OWT SHM. This method can identify micro-scale failure mechanisms within the turbine, including monitoring the formation of internal cracks and high-frequency stress waves generated by friction or other dynamic processes [101]. AE sensors are highly sensitive to minor changes, enabling the detection of developing faults before they escalate into severe issues. Additionally, AE detection serves as a non-destructive testing alternative, allowing for real-time monitoring from remote locations [102].

In the context of wind energy equipment, AE detection is particularly useful for monitoring critical components such as the tower, blades, and foundations. Joosse PA et al. [103,104] utilized acoustic emission technology to detect sounds associated with crack mechanisms, pinpointing damaged areas caused by blade cracking. Tziavos N et al. [105] applied AE technology to analyze damage evolution and failure mechanisms in grouted connections of Offshore Wind Turbines, subsequently calculating the potential damage under various complex stress states.

By strategically installing AE sensors, operators can gain insights into structural health, facilitating timely maintenance interventions. This proactive approach not only enhances safety but also extends the operational lifespan of the turbines.

#### 3.1.3. Strain Measurement for Wind Turbine SHM

Strain measurement is also a critical technology for detecting micro-scale changes at predetermined locations within structural health monitoring, essential for assessing the structural integrity of wind turbines under various complex loading conditions, including the weight of the wind turbine tower and upper structures, as well as harsh wave and wind loads [107,108,109]. In Offshore Wind Turbines, strain sensors are employed to evaluate the performance of key components such as the tower, rotor blades, and foundations. Typically, resistive or optical strain gauges are used to monitor localized deformations in offshore wind structural components, with sensors installed at specific locations on the OWT blades where significant deformation occurs [110,111].

Li [112] conducted a study using inverse finite element methods to monitor and analyze strain data from wind turbine towers under static and dynamic loading conditions. This analysis compared the strain data with Von Mises stress results to determine the optimal positioning of strain sensors on OWTs. Yi [113] developed an SHM system that integrated strain gauges with accelerometers and inclinometers for long-term monitoring of offshore structures, capturing dynamic response characteristics, including acceleration, dynamic tilt, and strain. Furthermore, research indicates that installing strain sensors on OWTs allows engineers to identify potential fault points and optimize designs to enhance performance, reducing operational and maintenance costs by 10% to 15% [109]. This information is crucial for understanding the long-term behavior of the structure and making informed decisions regarding maintenance and repairs [114]. In summary, we briefly summarize the different sensing techniques used for structural health monitoring of Offshore Wind Turbines as shown in the following figure.

### 3.2. Data Transmission Technologies SHM of OWTs

**Figure 5:**
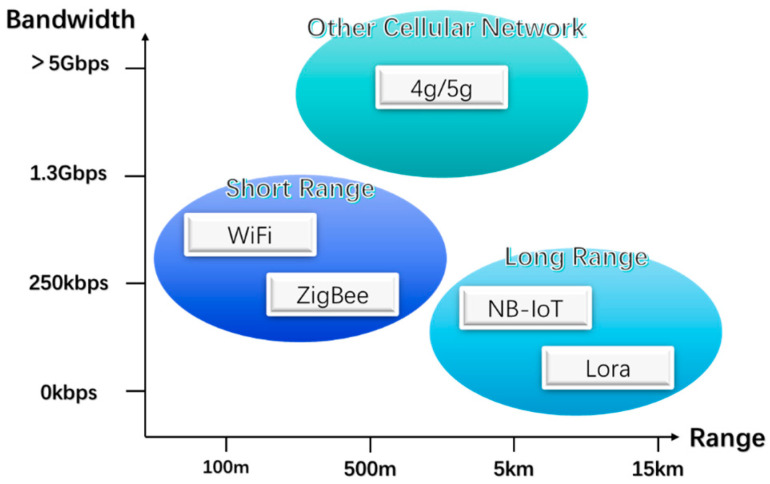
Diagram illustrating the bandwidth and transmission range of common wireless data transmission methods, including LoRa, Wi-Fi, NB-IoT, 4G/5G, and Zigbee [122].

Once data is collected from the wired cables using sensing technologies, the next step is to transmit this data to a central monitoring system. The key to the normal operation of the SHM system supporting wind turbine operations is real-time, accurate, and secure data transmission. This is achieved by constructing a data communication network using either wired or wireless transmission methods, enabling the monitoring of construction working conditions, timely detection of structural damage, and assessment of building safety [119]. Generally, traditional fiber optic or copper cable connections are suitable for data transmission over short distances due to their high reliability; however, their offshore applications are limited, and the installation and maintenance costs are high [120,121]. Therefore, the current data transmission technologies applied in SHM primarily utilize wireless communication technologies such as LoRa, Wi-Fi, NB-IoT, 4G/5G, and Zigbee [122]. Figure 5 illustrates the bandwidth and transmission range of various common wireless data transmission methods. Table 4 shows the performance comparison of various wireless transmission technologies for OWTs.

#### 3.2.1. Wi-Fi and Zigbee

ZigBee technology can be used to transmit structural health monitoring data to facilitate the monitoring of offshore wind power [127,128]. A comprehensive real-time online monitoring platform for offshore wind power has been proposed, which constructs a ZigBee wireless local area network through vibration sensors, collection nodes, and coordinator nodes for data transmission [124]. Wi-Fi is another widely used data transmission technology in SHM systems, offering high data transmission rates suitable for applications that require real-time monitoring and analysis [129,130]. In offshore wind farms, Wi-Fi networks can be established to connect sensors and data acquisition systems to local servers or cloud platforms. Wang [131] designed a dual-layer wireless sensor network for structural health monitoring that combines the advantages of ZigBee and Wi-Fi performance. The lower layer network uses low-power ZigBee protocol, while the upper layer network employs high-speed Wi-Fi communication for long-distance data transmission, enabling timely warnings in the event of sudden incidents affecting structural safety. Although Zigbee and Wi-Fi have advantages in offshore wind structure health monitoring [123,132], supporting higher bandwidth data transmission or transmission speeds, they also have disadvantages such as short transmission distances and high-power consumption, making them inconvenient for standalone use in offshore wind farms. They are typically used in conjunction with other long-distance communication technologies to enhance transmission distance [133,134].

#### 3.2.2. LoRa and NB-IoT

LoRa is a low-power, long-range wireless communication technology, particularly suitable for SHM applications in remote areas such as offshore wind farms [125,126]. It can transmit data over distances exceeding 10 km, making it ideal for connecting multiple sensors distributed throughout the wind farm. One of LoRa’s main advantages is its low power consumption, allowing sensors to operate for extended periods on battery power [135]. This is particularly beneficial for offshore applications that are difficult to maintain and costly. Furthermore, LoRa can penetrate obstacles, meaning data can be transmitted even in harsh marine environments [136]. NB-IoT is a new narrowband cellular communication low-power technology designed for low-power, wide-area applications [125,137]. NB-IoT can be directly deployed on GSM, UMTS, or LTE networks, transmitting data from SHM sensors in wired cables to central monitoring systems [138]. NB-IoT has several advantages, including expanded coverage, improved penetration in challenging environments, reduced costs, and enhanced performance [139]. Gliga L I [140] conducted a comprehensive comparison of various wireless communication technologies, including LoRa and NB-IoT, in wind turbine applications, considering both long-distance low-power protocols and short-distance high-speed protocols, and introduced a LoRa-based independent communication system architecture for wind farms.

In summary, many data transmission technologies are currently applied in offshore wind structural health monitoring, each with its own advantages and disadvantages. We briefly summarize the number of published papers related to these technologies in recent years in the table below.

### 3.3. Conclusion of Data Acquisition and Transmission Technology for SHM of Offshore Wind Turbines

Based on the contents of Table 4, we summarize the performance comparison of several commonly used wireless transmission technologies in offshore wind farm applications, which provides a reference for choosing a suitable data transmission scheme. We discuss the differences of different wireless transmission technologies in terms of transmission distance, bandwidth and anti-jamming ability. ZigBee and Wi-Fi have shorter transmission distances of 500 m and 250 m, but higher bandwidths of 10–250 kbps and 1.3 Gbps, respectively. They have weaker anti-jamming ability. LoRa and NB-IoT have farther transmission distances of up to 13 km and 10 km, with lower bandwidths of 0.3–37.5 kbps and 160–250 kbps, respectively, but they are more resistant to interference [141].

In offshore wind turbines, latency and interference issues are particularly acute due to complex environmental factors (wind speed variations, ocean fluctuations, etc.), and wireless sensor nodes often rely on batteries or other energy sources for power supply. Excessive power consumption may result in the need for frequent battery replacements for the sensors, which increases maintenance costs [142].

Different wireless transmission technologies are suitable for different application scenarios. ZigBee and Wi-Fi are suitable for short-distance and high-bandwidth applications, such as transmission inside wind farms. LoRa and NB-IoT are suitable for long-distance, low-power applications, such as data transmission within the entire wind farm [134]. In the structural health monitoring of offshore wind farms, it is necessary to consider the transmission distance, bandwidth and anti-interference ability and other factors; LoRa or NB-IoT can be used as the main long-distance transmission technology, with ZigBee or Wi-Fi as the short-distance transmission technology, to build a multi-level wireless transmission network.

Overall, this chapter explores in detail the main sensing and data transmission techniques used in structural health monitoring (SHM) of Offshore Wind Turbines (OWTs). The main sensing technologies include vibration monitoring, acoustic emission detection, and strain measurement, with the former allowing real-time monitoring of structural response through modal analysis and vibration pattern recognition; acoustic emission technology enabling the detection of internal micro-cracks and the timely detection of faults; and strain measurement focusing on the performance assessment of critical components. This chapter also discusses the data transmission technologies used for offshore wind SHM, comparing the advantages, disadvantages, and application characteristics of wireless transmission technologies such as Wi-Fi, Zigbee, LoRa, and NB-IoT, to ensure that the structural health monitoring data can be transmitted in real time to improve the safety and sustainability of Offshore Wind Turbines.

## 4. Structural Load Identification and Safety Evaluation for SHM in OWTs

### 4.1. Structural Load Identification for SHM in OWTs

#### 4.1.1. The Load Components of OWTs

**Figure 6:**
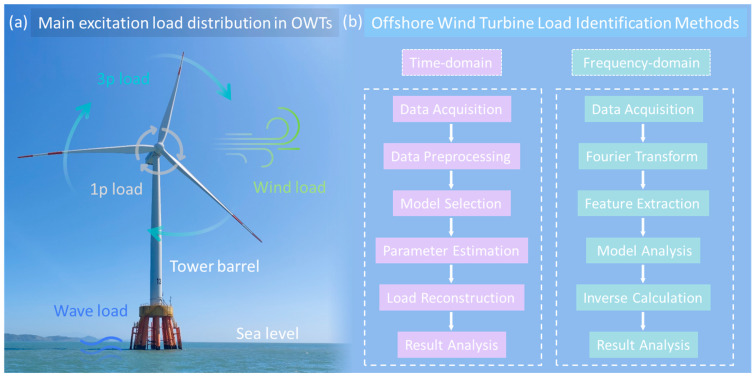
Structural Load Identification for SHM in OWTs. (**a**) The schematic representation of the load components acting on Offshore Wind Turbines (OWTs), including aerodynamic wind loads (blade thrust loads), wave loads, as well as 1p and 3p loads [147,148]. (**b**) Flowchart illustrating time-domain and frequency-domain methods for load identification in Offshore Wind Turbines (OWTs) [149,150].

The loads experienced by a wind turbine throughout its operational life predominantly consist of four key components: aerodynamic wind loads (blade thrust loads), wave loads, 1p loads, and 3p loads, as depicted in Figure 6a [143,144,145,146,147,148]. Aerodynamic wind loads, generated by the wind thrust acting on the blades and tower, include cyclic components that are largely influenced by the wind turbulence at the turbine site and the turbine’s operational parameters. Wave loads arise from the dynamic forces exerted by waves on the structural elements in contact with water. The 1p load refers to a cyclic vibrational load caused by mass and aerodynamic imbalances of the rotor at hub height, with a frequency corresponding to the rotor’s rotational speed. In contrast, the 3p load is induced by the blade shadow effect, where the blades intermittently pass in front of the tower, casting a shadow that reduces the thrust force acting on it.

#### 4.1.2. Time-Domain Methods for Identifying Loads in OWTs

In the field of offshore wind turbine load identification, both time-domain and frequency-domain approaches have been explored. Figure 6b depicts a flowchart illustrating the time-domain and frequency-domain methods for load identification in Offshore Wind Turbines (OWTs). One of the earliest applications of time-domain methods for identifying loads in offshore wind turbine structures was conducted by Klinkov and Fritzen [149,150,151,152,153]. They developed a joint input-state estimation algorithm to reconstruct unknown loads and validated the method’s accuracy through experiments using a physical model. Additionally, they examined the impact of noise on the accuracy of the load identification process [151,152,153]. This approach was successfully implemented for the online load identification of a 5 MW wind turbine in Germany, where strain and acceleration responses were used to inversely estimate the turbine’s top load. The results were then compared with blade thrust values derived from Betz’s theory [149,150], effectively addressing the challenge of operational load identification in wind turbine structures. Lourens et al. [154] introduced an advanced joint input-state estimation algorithm, relying on limited acceleration response measurements and employing linear minimum variance unbiased estimation. Notably, this method did not require assumptions about load inputs or regularization. Swartz et al. [155] employed wireless sensors to autonomously gather signals and identify the modal parameters of turbines. Using these identified parameters, they applied a time-domain method to determine wind loads and validated their findings with a physical model. They verified the method’s accuracy using a 6 MW finite element model, successfully estimating wave loads at the top of the tower and strain at the midline [156], demonstrating the efficacy of this approach in assessing hydrodynamic loads and the stiffness properties of support structures. While time-domain methods have proven effective in online load identification for Offshore Wind Turbines, their application in modal space involves complex and computationally intensive convolution relationships [157]. Moreover, many time-domain methods are recursive, making them sensitive to initial conditions and susceptible to cumulative errors. These methods also exhibit limitations in noise resistance and robustness when applied to real-world engineering scenarios.

#### 4.1.3. Frequency-Domain Methods for Identifying Loads in OWTs

Bartlet and Flannelly [158] pioneered the use of acceleration data for identifying loads at the main rotor shaft of helicopters, marking the advent of frequency-domain load identification techniques. Following this, Rebelo et al. [159] applied similar principles to an 80-m-high wind turbine structure by installing four layers of accelerometers and strain sensors. By analyzing the recorded vibration response signals, they identified the turbine’s modal parameters and constructed an inverse model to estimate the wind loads acting on the turbine system. Researchers from the German National Renewable Energy Laboratory, including Pahn and colleagues [160,161,162], further advanced frequency-domain methods by employing the inverse matrix approach of the transfer function to reconstruct dynamic loads on wind turbines. This method accounted for the coupling effects between the blades and the tower. Dynamic loads were derived from acceleration responses using the inverse transfer function matrix, while static loads were determined through strain responses. The total load acting on the tower was calculated by summing these static and dynamic loads. This technique was successfully applied to a 5 MW wind turbine structure with a jacket foundation at the Bremerhaven wind farm in Germany for load identification. Despite the simplicity of frequency-domain identification principles, this approach is not well-suited for non-stationary vibrations or scenarios with limited sample sizes. Moreover, the method suffers from ill-posedness near the structure’s natural frequencies due to its computational characteristics. Cosack [163], leveraging operational parameters such as rotor speed, pitch angle, and generated power, utilized neural networks as a substitute for traditional transfer function matrices to model the relationship between a wind turbine’s structural inputs and its loads. This approach enabled the estimation of fatigue loads, offering a novel methodology for load prediction. Noppe et al. [164,165,166] further explored the use of neural network algorithms to estimate blade thrust loads based on Supervisory Control and Data Acquisition (SCADA) data. Their method was validated against both simulation results and real-world offshore wind turbine measurements. Under normal operating conditions, the neural network approach demonstrated a relative error of approximately 15%, highlighting its potential for practical applications in load estimation and turbine monitoring.

### 4.2. Safety Evaluation for SHM in OWTs

The operational conditions of offshore wind turbine structures throughout their service life are highly complex. In addition to standard working loads, such as those caused by wind, waves, and currents, these structures are also subjected to environmental extremes, including earthquakes and typhoons, as well as accidental loads from events such as ship collisions, fires, and explosions. The combined influence of these diverse loading conditions makes offshore wind turbine foundations particularly vulnerable to fatigue damage, corrosion, and other forms of structural degradation. In severe cases, these issues can escalate to structural failure, leading to safety incidents with potentially significant human and financial consequences. Therefore, conducting comprehensive safety assessments of Offshore Wind Turbines is critical for developing effective risk prevention.

#### 4.2.1. Failure Modes of OWTs

**Figure 7:**
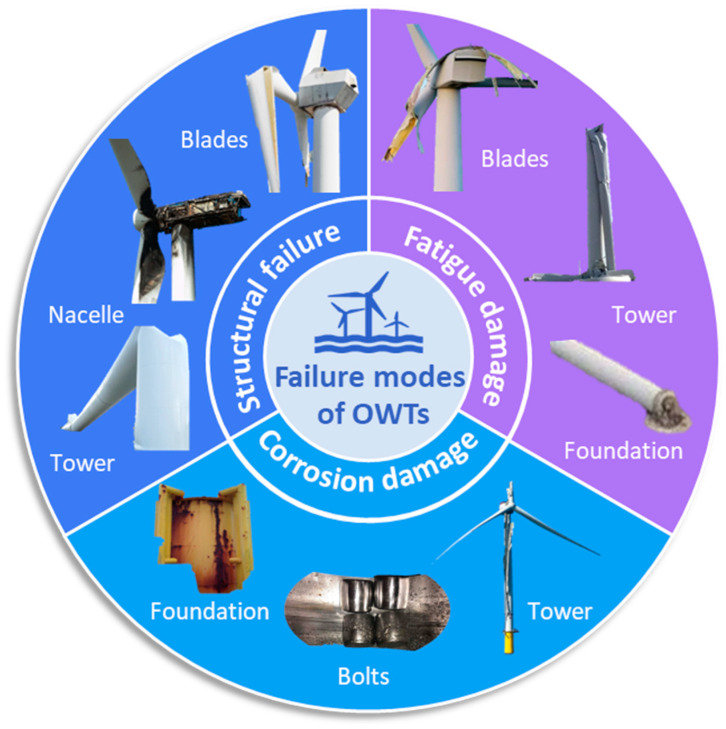
The failure modes of offshore wind turbine structures throughout their service life include structural failure, fatigue damage, and corrosion damage [165,166,167,168,169,170,171,172].

The main causes of failure for monopile and bucket foundations of Offshore Wind Turbines during their service life can be categorized into three key areas [166,167,168,169]. Structural failure results from excessive loads or material defects, causing cracking or deformation, with damage commonly occurring in the blades, tower, nacelle, and bucket foundation. Fatigue damage arises from repeated cyclic loading due to wind, waves, and currents, progressively weakening components like the blades, tower, and foundation. Lastly, corrosion damage is driven by the harsh marine environment, where seawater accelerates material degradation, particularly affecting the tower, bolts, and foundation, leading to compromised structural integrity and performance. The failure modes of offshore wind turbine structures throughout their service life are depicted in Figure 7.

#### 4.2.2. Safety Evaluation Methods for OWTs

With the continuous advancements in offshore wind turbine structural health monitoring (SHM) technologies and finite element simulation techniques, it has become increasingly feasible to identify structural modal parameters using prototype observation data. Bajri et al. [170] developed an error measurement and covariance-driven automated operational modal identification system, which uses vibration response data collected during turbine shutdowns to validate its effectiveness in assessing structural safety. Similarly, Benedetti et al. [171] proposed using the strain difference between adjacent strain gauges as an indicator for tower safety evaluation, while Ziegler et al. [172] calculated damage-equivalent loads using strain gauges on the support structure, achieving a monthly prediction error of less than 4%. Yi et al. [113] implemented a system for jacket foundations using strain, acceleration, and inclinometer sensors, applying the least-squares frequency domain method to identify natural frequencies and damping. Hu et al. [173] proposed a strain-based approach for long-term dynamic modal analysis, using it to evaluate the safety of a 5 MW wind turbine during extended operation. Liu et al. [174] introduced a time-frequency analysis method based on signal modal functions to further enhance the identification of offshore wind turbine modal parameters. Smarsly and Adams [107,175] refined finite element models using prototype monitoring data, creating a damage database based on modal responses under various damage conditions. This database serves as a damage evaluation tool for wind turbine structures.

Devriendt [115] and Oliveira [176,177] developed automated modal identification systems that track dynamic changes in support structures, facilitating ongoing safety evaluations of wind turbine infrastructure. Tang et al. [178] used inclinometers and accelerometers to capture the dynamic response of monopile foundations, developing a scour warning system and defining scouring thresholds. Yu et al. [179] investigated the impact of wave-current interactions on scour development around bucket foundations, revealing that the largest scour pits form under these interactions. Opoka et al. [180] and Mieloszyk et al. [181] conducted experiments using a physical model of a tripod wind turbine, gathering stress response data from the structure under both damaged and undamaged conditions. By analyzing the differences in frequency domain responses before and after damage, they established four distinct damage indicators. These indicators were designed to enable the detection and localization of structural damage, offering valuable tools for monitoring and maintaining the integrity of wind turbine systems.

### 4.3. Conclusion of Structural Load Identification and Safety Evaluation for SHM in OWTs

Structural load identification and safety evaluation are essential for structural health monitoring (SHM) in Offshore Wind Turbines (OWTs) to ensure their long-term reliability. Key load components include wind, wave, 1p, and 3p loads, identified through time-domain and frequency-domain methods. Time-domain approaches use acceleration and strain responses for online load estimation but are prone to noise and cumulative errors. Frequency-domain methods, relying on inverse transfer functions, face limitations near natural frequencies. Safety evaluation focuses on structural failure, fatigue, and corrosion, with vibration responses and finite element models aiding in damage detection and long-term monitoring.

## 5. Conclusions and Future Outlook

In the offshore wind power sector, future material design for blades and supporting structures will be crucial for improving turbine performance and reliability. Wind turbine blades must demonstrate exceptional strength, toughness, and fatigue resistance, typically achieved through laminated composites like glass and carbon fibers, which enhance load capacity while reducing weight. The rise of bio-composites is also notable, as they aim to meet performance standards with reduced environmental impact. However, traditional uniaxial stress testing is insufficient for assessing composite materials under real-world conditions, making the development of new testing methods and precise micromechanical models a research priority. For supporting structures, concrete, steel, and composites are primary choices. Concrete is suitable for gravity-based foundations due to its durability, but its weight and cost limit its use in deep-water environments. Steel, known for its high strength and low weight, is ideal for monopile and multi-leg structures, although corrosion resistance remains a challenge. The emergence of floating wind turbines provides a promising solution for deep-water energy, utilizing a combination of steel, concrete, and high-performance composites to enhance stability and durability.

Table 5 summarizes the main points of this review and reports on the main issues and application challenges and future trends in material design and structural health monitoring for offshore wind. It should be noted that this discussion focuses on offshore horizontal axis wind turbines and does not include vertical axis wind turbines. We point out that although existing technologies have made some progress in improving the reliability and safety of offshore wind turbines, they are still facing problems in terms of corrosion damage of their materials, monitoring accuracy and real-time performance, optimization of load assessment algorithms, and integration of intelligent monitoring systems.

In response to the specific challenges of offshore wind power, researchers are developing adaptive sensors for structural health monitoring (SHM) of Offshore Wind Turbines. These sensors employ various wireless technologies to collect data and convert it into digital signals for analysis, accurately measuring displacement, vibration, stress, strain, and temperature. To ensure reliable data transmission, engineers are optimizing communication protocols and network topologies to enhance stability and efficiency. Future SHM systems are expected to integrate advanced miniaturized sensors and increasingly depend on wireless sensor networks (WSNs) to reduce wiring complexity and improve data collection flexibility. The integration of machine learning and artificial intelligence will further enhance the efficiency of data acquisition and transmission, paving the way for more robust monitoring solutions. In addition, with the popularity of offshore wind turbines, commercial application will become an important direction for research. The design selection of wind turbine materials and the use of structural health monitoring systems require high costs, and future research should focus on reducing the cost of monitoring, improving the integration of the system, and promoting the practical application of the technology.

With the rapid advancement of computer technology, artificial intelligence algorithms have emerged as powerful tools for addressing complex engineering challenges. By analyzing various operational parameters—such as rotor speed, pitch angle, and power output—artificial neural networks can effectively model the intricate relationships between structural inputs and the loads acting on the turbine. Furthermore, by recognizing patterns in load data and correlating them with the turbine’s operational state, potential issues can be identified before they lead to failure. This proactive approach enhances the reliability of wind turbines and contributes to optimizing maintenance schedules, ultimately reducing downtime and operational costs.

Overall, material design in offshore wind power faces the dual tasks of improving performance requirements and addressing environmental challenges. Future developments will focus on optimizing material selection, innovating design methods, and overcoming challenges posed by various environmental conditions to advance and apply offshore wind power technology. The SHM technologies for Offshore Wind Turbines are advancing rapidly, with a trend toward greater intelligence, connectivity, precision, and automation, aiming to meet the growing demands of the wind power market and ensure the long-term stability of wind energy facilities. In the future, artificial intelligence and machine learning will be integrated into the operational framework of Offshore Wind Turbines, improving their performance, safety, and efficiency through innovative load identification and safety assessment methods.

## Figures and Tables

**Table 1:** Comparison with Existing Offshore Wind Structural Health Monitoring Review Papers (data obtained from Web of Science, from 2015 to 2024, keywords: OWTs, SHM, materials, sensors, etc.).

Ref.	Description and Contribution	Limitations
[21]	This article reviews the current state of commercial and research health monitoring systems, with a focus on their application to key wind turbine components and the associated challenges.	While offshore wind farms and data acquisition systems are mentioned, the analysis of unique challenges such as harsh environmental conditions, corrosion, and data transmission is not comprehensive.
[22]	This article examines advanced signal processing and machine learning techniques for structural health monitoring (SHM) and condition monitoring (CM) in detecting gearbox and blade damage in wind turbines. Additionally, it provides an initial investigation into data acquisition systems for offshore wind farms.	This article highlights the diversity of SHM methods but does not provide a standardized framework for their implementation, making it difficult to compare and integrate different approaches.
[23]	This article covers a systematic study and summary of health monitoring systems and operational safety assessment techniques for offshore wind power structures.	This article focuses primarily on theoretical models and simulations that are not adequately validated by real-world offshore wind materials structural design, etc., thus limiting their practical applicability.
[24]	This article covers current damage identification techniques for wind turbine towers and foundations, in addition to providing insights into the challenges of wind turbine monitoring and highlighting potential avenues for future development.	This article mentions potential avenues for development but lacks detailed insights into specific areas of future research, particularly in handling structural loads and enhancing sensing technologies.
[25]	This article covers state-of-the-art knowledge on methods and approaches to handling structural loads, with a focus on offshore wind turbine systems and applied sensing technologies.	This article is narrowly focused on specific types of wind turbine structures and does not address the broader range of OWT support structures.
[26]	This article covers an extensive review of the last 20 years of non-destructive methods related to structural health monitoring and condition monitoring of wind turbine structures.	This article focuses on the past 20 years of non-destructive methods, which may lead to an underrepresentation of the most recent advances and trends in health monitoring systems.

**Table 2:** Summary of design, materials, and protection for OWTs.

Components	Design Considerations	Materials	Protection Methods	References
Rotor	Blade thickness Blade dimensions Bending loads on the blade Wind load design	Glass fiber, Carbon fiber, Biological fibers, Polymers	Paint coatings Metallic coatings	[27,88,89,90]
Nacelle	Insulation Heat dissipation	Iron, Low alloy steel, High alloy steel		
Tower	Load-bearing capacity Structural stability Wind load resistance	Steel, Hybrid, Concrete	Paint coatings Metallic coating’s Corrosion allowance Cathodic protection	[73,82,89]
Foundation	Load-bearing capacity Vibration isolation Corrosion protection	Steel, Concrete	Paint coatings Cathodic protection	[82,89]

**Table 4:** Performance comparison of various wireless transmission technologies for OWTs.

Technology	Type	Range	Bandwidth	Anti-Interference
ZIGBEE [123]	WLAN (Wireless Local Area Network)	<500 m	10–250 kbps	Weak
WI-FI [124]		<250 m	1.3 Gbps	Weak
LORA [125,126]	LPWAN (Low-Power Wide-Area Network)	Up to 13 km	0.3–37.5 kbps	Strong
NB-IOT [126]		Up to 10 km	160–250 kbps	Strong

**Table 5:** Future Research Directions in Material Selection and Structural Health Monitoring for Offshore Wind Turbines.

Existing Challenges	Future Research Directions
Material selection and Corrosion of offshore wind structures (blades, foundations) issues	Development of new corrosion-resistant materials to enhance the service life of turbine materials; Bio-based composites also show good prospects for development; Closed-loop recycling of end-of-life wind turbines or extending the operational lifetime of wind turbines [89].
Real-Time and Accuracy Issues in Offshore Wind Structural Health Monitoring Technology	Fault Diagnosis Methods Integrating Vibration, Models and Artificial Intelligence; Use different wireless communication protocols to build a multi-level wireless transmission network to meet the needs of different application scenarios [24]; Optimize the communication protocol and network topology to improve the stability and efficiency of data transmission.
Load Evaluation Algorithm Optimization	Combining machine learning and artificial intelligence techniques to improve the efficiency of data analysis in the prediction of wind turbine health status and fault diagnosis [182].
Integration issues of intelligent monitoring systems	Integrate Internet of Things (IoT) technology and Artificial Intelligence (AI) technology to realize multi-sensor data fusion and intelligent decision support.

## Data Availability

Data availability is not applicable to this article as no new data were created or analyzed in this study.
